# Supplementary material for: A Marked Low-Grade Inflammation and a Significant Deterioration in Metabolic Status in First-Episode Schizophrenia: A Five-Year Follow-Up Study
Source: Metabolites. 2022 Oct 17;12(10):983. doi: 10.3390/metabo12100983 (PMC9610466; doi:10.3390/metabo12100983)
Supplement: Supplementary file 1 [file metabolites-12-00983-s001.zip › metabolites-1968400-supplementary.pdf]

Supplementary Material

A Marked Low-grade Inflammation and a Significant Deterioration in Metabolic Status in First Episode Schizophrenia: a Five-Year Follow-up Study

Madis Parksepp[1,2*], Liina Haring[1,3,4], Kalle Kilk[4], Egon Taalberg[4], Raul Kangro[5], Mihkel Zilmer[4], Eero Vasar[4]

[1] Institute of Clinical Medicine University of Tartu; Psychiatry Clinic of Tartu University Hospital, Estonia
[2] Psychiatry Clinic of Viljandi Hospital, Estonia; Madis.Parksepp@vmh.ee (corresponding author*)
[3] Psychiatry Clinic of Tartu University Hospital, Estonia; Liina.Haring@kliinikum.ee
[4] Institute of Biomedicine and Translational Medicine, Centre of Excellence for Genomics and Translational Medicine, University of Tartu, Estonia; Kalle.Kilk@ut.ee; Egon.Taalberg@ut.ee; Mihkel.Zilmer@ut.ee; Eero.Vasar@ut.ee
[5] Institute of Mathematics and Statistics, University of Tartu, Estonia; Raul.Kangro@ut.ee

Table S1. Serum mean levels, standard deviations (SD), and range of inflammatory and metabolic protein biomarkers (pg/ml), acylcarnitines, hexoses, body mass index, and waist circumference for control subjects (CSs, n = 58), first-episode psychosis (FEP) patients at baseline (before treatment with antipsychotics, FEP_{b}, n = 54), after 0.6-year treatment ($FEP_{0.6\text{-year}}$, n = 47), and after 5.1-year treatment ($FEP_{5.1\text{-year}}$, n = 38) with antipsychotics. In comparison between reduced and unrestricted models, *p*-values have been multiple tests corrected according to the false discovery rate (FDR) method. The unrestricted regression model is designated as True (i.e. more complex set of predictor variables explained more effectively biomarker level alterations over time) or False (i.e. unrestricted model did not provide more explanatory power than a simple one).

Regression equations for estimating biomarker level or biomarkers ratio alterations.	
Reduced model	log(biomarker)~Age+Gender+Smoking+(1|Patient)
Unrestricted model	log(biomarker)~Age+ Gender+Smoking+Visit+Time difference+(1|Patient)

Visit = biomarker measurements at three-time points in the patients' group, Time1 = time difference between FEP_{b} and $FEP_{0.6\text{-year}}$, Time2 = time difference between $FEP_{0.6\text{-year}}$ and $FEP_{5.1\text{-year}}$, (1|Patient) = random effects of patients.

	**CSs**<br>**Mean ± SD**<br>**Median (range)**	**FEP_{b}**<br>**Mean ± SD**<br>**Median (range)**	**$FEP_{(0.6\text{-year})}$**<br>**Mean ± SD**<br>**Median (range)**	**$FEP_{(5.1\text{-year})}$**<br>**Mean ± SD**<br>**Median (range)**	**Comparison between models**	
					**Adjusted *p*-value**	**True (T) or false (F)**
Body mass index (BMI), waist circumference						
BMI	22.6 ± 2.78<br>22.6 (16.8 – 28.9)	22.8 ± 3.00<br>22.2 (18.4 – 30.2)	25.3 ± 3.94<br>24.4 (18.8 – 34.7)	27.8 ± 4.71<br>27.5 (18.8 – 43.0)	6×10^{-12}	T
Waist circumference	77.9 ± 12.6<br>77.0 (61.0 – 108)	81.0 ± 9.89<br>79.5 (64.0 – 110)	86.3 ± 11.2<br>86.0 (68.0 – 114)	94.4 ± 11.2<br>96.5 (65.0 – 118)	6×10^{-11}	T

Continued.

	CSs<br>Mean ± SD<br>Median (range)	FEP_b<br>Mean ± SD<br>Median (range)	$FEP_{(0.6\text{-year})}$<br>Mean ± SD<br>Median (range)	$FEP_{(5.1\text{-year})}$<br>Mean ± SD<br>Median (range)	Comparison between models	
					Adjusted<br>*p*-value	True (T) or<br>false (F)
Inflammatory biomarkers						
Interleukin (IL-)2	2.27 ± 1.26<br>2.50 (0.66 – 6.28)	3.15 ± 0.87<br>3.06 (1.18 – 6.69)	2.39 ± 1.14<br>2.10 (1.14 – 7.90)	3.52 ± 1.33<br>3.60 (0.72 – 8.27)	5×10^{-7}	T
IL-4	1.33 ± 0.51<br>1.26 (0.79 – 3.71)	1.87 ± 0.57<br>1.79 (1.02 – 3.75)	1.62 ± 0.63<br>1.41 (0.92 – 4.32)	2.21 ± 0.56<br>2.24 (0.82 – 3.47)	3×10^{-9}	T
IL-6	0.76 ± 0.59<br>0.59 (0.15 – 2.91)	1.27 ± 1.14<br>0.87 (0.33 – 5.03)	0.93 ± 0.76<br>0.72 (0.19 – 4.16)	1.12 ± 0.74<br>0.90 (0.52 – 4.45)	6×10^{-4}	T
IL-8	5.98 ± 2.90<br>5.61 (1.61 – 13.8)	6.58 ± 4.07<br>5.59 (1.72 – 17.9)	5.66 ± 3.88<br>4.30 (1.25 – 17.4)	7.05 ± 3.05<br>6.59 (2.63 – 14.1)	4×10^{-4}	T
IL-10	0.71 ± 0.42<br>0.59 (0.19 – 2.11)	0.73 ± 0.37<br>0.73 (0.19 – 1.65)	0.75 ± 0.49<br>0.65 (0.27 – 2.29)	1.44 ± 0.93<br>1.13 (0.50 – 4.94)	4×10^{-7}	T
IL-1α	0.19 ± 0.13<br>0.14 (0.060 – 0.61)	0.29 ± 0.23<br>0.23 (0.060 – 1.33)	0.23 ± 0.19<br>0.17 (0.07 – 1.26)	0.30 ± 0.11<br>0.31 (0.060 – 0.58)	2×10^{-4}	T
IL-1β	1.63 ± 0.91<br>1.48 (0.45 – 4.47)	1.41 ± 0.66<br>1.37 (0.44 – 3.44)	1.37 ± 0.76<br>1.39 (0.39 – 3.39)	1.60 ± 0.47<br>1.66 (0.46 – 2.23)	1×10^{-1}	F
Interferon gamma (IFN-γ)	0.30 ± 0.090<br>0.27 (0.19 – 0.55)	0.36 ± 0.14<br>0.36 (0.19 – 0.92)	0.27 ± 0.12<br>0.22 (0.17 – 0.69)	0.44 ± 0.21<br>0.38 (0.16 – 1.14)	3×10^{-8}	T
Tumor necrosis factor alpha (TNF-α)	2.22 ± 1.40<br>1.86 (0.86 – 8.70)	1.98 ± 0.84<br>1.83 (0.97 – 5.76)	1.91 ± 0.59<br>1.79 (1.03 – 3.59)	2.39 ± 0.79<br>2.32 (0.90 – 4.61)	3×10^{-3}	T
Monocyte chemoattractant protein-1 (MCP-1)	131 ± 76.7<br>115 (23.1 – 365)	127 ± 77.4<br>111 (21.2 – 381)	135 ± 80.2<br>116 (10.6 – 344)	170 ± 72.1<br>159 (36.5 – 353)	4×10^{-3}	T
Ferritin	43.6 ± 55.2<br>21.4 (1.22 – 255)	90.0 ± 82.5<br>62.0 (4.26 – 386)	64.3 ± 75.8<br>32.5 (5.09 – 342)	92.7 ± 77.8<br>77.4 (3.35 – 269)	3×10^{-3}	T
Metabolic biomarkers						
Resistin	2.53 ± 0.57<br>2.51 (0.88 – 4.10)	3.15 ± 1.33<br>2.85 (0.75 – 8.58)	2.78 ± 0.88<br>2.73 (1.42 – 5.63)	2.18 ± 1.01<br>1.90 (1.13 – 6.40)	1×10^{-4}	T
Insulin	15.6 ± 14.3<br>9.02 (1.80 – 67.6)	12.9 ± 14.8<br>7.24 (1.54 – 64.4)	18.3 ± 16.0<br>12.3 (2.41 – 66.8)	8.28 ± 6.59<br>7.30 (1.90 – 35.6)	2×10^{-2}	F
Leptin	2.07 ± 2.18<br>1.34 (0.38 – 12.8)	1.40 ± 1.69<br>0.87 (0.000 – 10.4)	2.59 ± 2.77<br>1.31 (0.000 – 12.5)	3.29 ± 3.90<br>1.39 (0.45 – 15.5)	1×10^{-4}	T
Plasminogen activator inhibitor-1 (PAI-1)	21.9 ± 9.56<br>20.5 (6.33 – 49.9)	29.7 ± 13.6<br>26.5 (2.13 – 61.7)	25.8 ± 14.9<br>20.9 (4.81 – 73.0)	20.4 ± 12.8<br>18.1 (0.95 – 63.2)	2×10^{-4}	T

Continued.

	CSs<br>Mean ± SD<br>Median (range)	FEP_b<br>Mean ± SD<br>Median (range)	$FEP_{(0.6\text{-}year)}$<br>Mean ± SD<br>Median (range)	$FEP_{(5.1\text{-}year)}$<br>Mean ± SD<br>Median (range)	Comparison between models	
					Adjusted *p*-value	True (T) or false (F)
Acylcarnitines						
C0	32.0 ± 7.82<br>31.8 (16.6 – 51.8)	34.6 ± 7.40<br>34.4 (18.3 – 52.0)	34.0 ± 9.63<br>33.4 (15.8 – 58.4)	37.1 ± 10.3<br>34.2 (18.7 – 67.2)	8×10^{-1}	F
C16	0.082 ± 0.021<br>0.080 (0.035 – 0.13)	0.11 ± 0.032<br>0.11 (0.046 – 0.20)	0.092 ± 0.029<br>0.085 (0.041 – 0.20)	0.11 ± 0.040<br>0.10 (0.057 – 0.22)	1×10^{-5}	T
C16-OH	0.009 ± 0.013<br>0.009 (0.000 – 0.084)	0.017 ± 0.005<br>0.016 (0.009 – 0.027)	0.019 ± 0.028<br>0.015 (0.008 – 0.20)	0.053 ± 0.099<br>0.022 (0.000 – 0.50)	3×10^{-2}	F
C16:1	0.026 ± 0.008<br>0.025 (0.015 – 0.071)	0.036 ± 0.012<br>0.036 (0.015 – 0.064)	0.028 ± 0.012<br>0.023 (0.011 – 0.062)	0.039 ± 0.022<br>0.032 (0.018 – 0.11)	4×10^{-5}	T
C16:1-OH	0.007 ± 0.013<br>0.008 (0.000 – 0.10)	0.014 ± 0.004<br>0.013 (0.007 – 0.028)	0.014 ± 0.012<br>0.011 (0.006 – 0.092)	0.034 ± 0.054<br>0.018 (0.000 – 0.24)	1×10^{-2}	F
C16:2	0.014 ± 0.007<br>0.012 (0.008 – 0.048)	0.015 ± 0.004<br>0.014 (0.008 – 0.027)	0.013 ± 0.005<br>0.013 (0.006 – 0.027)	0.026 ± 0.025<br>0.019 (0.008 – 0.11)	2×10^{-5}	T
C16:2-OH	0.016 ± 0.015<br>0.021 (0.000 – 0.045)	0.027 ± 0.005<br>0.027 (0.018 – 0.038)	0.029 ± 0.009<br>0.028 (0.019 – 0.075)	0.027 ± 0.018<br>0.024 (0.000 – 0.085)	2×10^{-3}	T
C18:0	0.039 ± 0.009<br>0.038 (0.022 – 0.073)	0.046 ± 0.013<br>0.046 (0.025 – 0.085)	0.042 ± 0.013<br>0.040 (0.022 – 0.074)	0.052 ± 0.021<br>0.043 (0.026 – 0.12)	3×10^{-3}	T
C18:1	0.096 ± 0.030<br>0.092 (0.058 – 0.18)	0.14 ± 0.046<br>0.14 (0.049 – 0.27)	0.096 ± 0.041<br>0.086 (0.039 – 0.27)	0.14 ± 0.048<br>0.13 (0.072 – 0.27)	8×10^{-8}	T
C18:1-OH	0.017 ± 0.018<br>0.015 (0.000 – 0.075)	0.028 ± 0.012<br>0.024 (0.013 – 0.053)	0.027 ± 0.016<br>0.017 (0.011 – 0.075)	0.031 ± 0.024<br>0.020 (0.000 – 0.10)	4×10^{-3}	T
C18:2	0.031 ± 0.009<br>0.029 (0.013 – 0.055)	0.042 ± 0.014<br>0.039 (0.020–0.081)	0.033 ± 0.015<br>0.030 (0.015 – 0.094)	0.047 ± 0.015<br>0.048 (0.025 – 0.093)	6×10^{-7}	T
C2	4.96 ± 1.68<br>4.90 (1.78 – 11.3)	4.65 ± 3.47<br>3.38 (0.51 – 14.0)	4.21 ± 1.70<br>3.95 (0.98 – 7.90)	6.97 ± 2.67<br>6.55 (3.16 – 13.2)	1×10^{-6}	T
C3	0.36 ± 0.11<br>0.36 (0.13 – 0.61)	0.25 ± 0.077<br>0.23 (0.14 – 0.49)	0.33 ± 0.13<br>0.30 (0.14 – 0.65)	0.54 ± 0.17<br>0.504 (0.28 – 1.00)	1×10^{-18}	T
C3-DC(C4-OH)	0.036 ± 0.023<br>0.043 (0.000 – 0.092)	0.062 ± 0.048<br>0.053 (0.025 – 0.32)	0.049 ± 0.017<br>0.045 (0.029 – 0.13)	0.050 ± 0.028<br>0.042 (0.000 – 0.12)	3×10^{-3}	T
C5-OH(C3-DC-M)	0.035 ± 0.032<br>0.049 (0.000 – 0.091)	0.060 ± 0.015<br>0.057 (0.036 – 0.11)	0.061 ± 0.015<br>0.059 (0.035 – 0.12)	0.062 ± 0.029<br>0.055 (0.000 – 0.15)	3×10^{-5}	T

Continued.

	CSs<br>Mean ± SD<br>Median (range)	FEP_b<br>Mean ± SD<br>Median (range)	$FEP_{(0.6\text{-}year)}$<br>Mean ± SD<br>Median (range)	$FEP_{(5.1\text{-}year)}$<br>Mean ± SD<br>Median (range)	Comparison between models	
					Adjusted<br>*p*-value	True (T) or<br>false (F)
Acylcarnitines						
C3-OH	0.028 ± 0.037<br>0.035 (0.000 – 0.25)	0.038 ± 0.010<br>0.040 (0.018 – 0.066)	0.043 ± 0.018<br>0.039 (0.023 – 0.14)	0.062 ± 0.060<br>0.044 (0.000 – 0.31)	2×10^{-2}	F
C3:1	0.018 ± 0.017<br>0.026 (0.000 – 0.069)	0.030 ± 0.007<br>0.030 (0.018 – 0.048)	0.036 ± 0.035<br>0.031 (0.019 – 0.26)	0.042 ± 0.029<br>0.035 (0.000 – 0.12)	6×10^{-3}	F
C4	0.22 ± 0.069<br>0.21 (0.12 – 0.42)	0.15 ± 0.043<br>0.15 (0.071 – 0.32)	0.17 ± 0.052<br>0.17 (0.077 – 0.31)	0.32 ± 0.11<br>0.31 (0.13 – 0.56)	1×10^{-15}	T
C4:1	0.026 ± 0.025<br>0.036 (0.000 – 0.089)	0.046 ± 0.009<br>0.046 (0.027 – 0.061)	0.051 ± 0.033<br>0.045 (0.030 – 0.26)	0.064 ± 0.038<br>0.060 (0.000 – 0.16)	2×10^{-5}	T
C5	0.17 ± 0.050<br>0.16 (0.000 – 0.32)	0.14 ± 0.042<br>0.14 (0.065 – 0.31)	0.17 ± 0.069<br>0.16 (0.075 – 0.39)	0.18 ± 0.067<br>0.18 (0.073 – 0.34)	4×10^{-4}	T
C5-M-DC	0.023 ± 0.021<br>0.031 (0.000 – 0.074)	0.034 ± 0.011<br>0.037 (0.014 – 0.059)	0.036 ± 0.013<br>0.036 (0.010 – 0.089)	0.030 ± 0.022<br>0.023 (0.000 – 0.10)	2×10^{-2}	F
C5:1	0.044 ± 0.040<br>0.056 (0.000 – 0.12)	0.076 ± 0.017<br>0.075 (0.040 – 0.14)	0.074 ± 0.021<br>0.071 (0.043 – 0.17)	0.091 ± 0.057<br>0.078 (0.000 – 0.28)	5×10^{-4}	T
C5:1-DC	0.015 ± 0.014<br>0.020 (0.000 – 0.053)	0.023 ± 0.006<br>0.025 (0.010 – 0.032)	0.024 ± 0.008<br>0.023 (0.010 – 0.053)	0.024 ± 0.017<br>0.019 (0.000 – 0.083)	1×10^{-2}	F
Hexoses, ratios of acylcarnitines						
Hexoses	4144 ± 823<br>4094 (2780 – 6663)	4258 ± 750<br>4075 (2802 – 5918)	4287 ± 867<br>4264 (2760 – 7189)	4887 ± 1104<br>4711 (3086 – 7271)	1×10^{-3}	T
(C16+C18)/C0	0.0040 ± 0.0013<br>0.0036 (0.0023 – 0.0092)	0.0049 ± 0.0016<br>0.0046 (0.0019 – 0.010)	0.0041 ± 0.0011<br>0.0037 (0.0025 – 0.0069)	0.0046 ± 0.0020<br>0.0041 (0.0020 – 0.010)	5×10^{-3}	T
(C16+C18:1)/C2	0.043 ± 0.015<br>0.038 (0.023 – 0.092)	0.085 ± 0.082<br>0.063 (0.022 – 0.50)	0.054 ± 0.047<br>0.042 (0.018 – 0.28)	0.037 ± 0.009<br>0.034 (0.019 – 0.056)	5×10^{-10}	T
(C2+C3+C4+C5)/C0	0.19 ± 0.091<br>0.18 (0.066 – 0.73)	0.16 ± 0.12<br>0.12 (0.024 – 0.55)	0.16 ± 0.086<br>0.16 (0.033 – 0.61)	0.22 ± 0.084<br>0.20 (0.11 – 0.44)	2×10^{-5}	T

Table S2. Estimated effects of the complex set of predictor variables on serum concentrations of inflammatory and metabolic protein biomarkers, acylcarnitines, hexoses, body mass index and waist circumference between control subjects (CSs, n = 58), first-episode psychosis patients at baseline (before treatment with antipsychotics, FEP_{b}, n = 54), after 0.6-year treatment ($FEP_{0.6\text{-year}}$, n = 47), and after 5.1-year treatment ($FEP_{5.1\text{-year}}$, n = 38) with antipsychotics: results from linear mixed-effects model.

	Intercept	Age	Gender	Smoking status	**Disease and Treatment Effect**			Time-Diff1	Time-Diff2
					**FEP patients before treatment**	**FEP patients after 0.6-year treatment**	**FEP patients after 5.1-year treatment**		
	**Effects of Independent Variables on the Dependent Variable (*F*-Value, *p*-Value)**								
					***t*-Value, *p*-Value**				
Body mass index (BMI), waist circumference									
BMI	$F_{(1,110)}$ = 1798, $p < 1 \times 10^{-4}$	$F_{(1,77)}$ = 2.83, p = 0.10	$F_{(1,110)}$ = 0.41, p = 0.52	$F_{(2,77)}$ = 0.39, p = 0.67	**$F_{(3,77)}$ = 26.88, $p < 1 \times 10^{-4}$**			$F_{(1,77)}$ = 1.29, p = 0.26	$F_{(1,77)}$ = 3.90, p = 0.05
					$t_{(77)}$ = -0.13, p = 0.90	**$t_{(77)}$ = 3.59, $p = 6 \times 10^{-4}$**	**$t_{(77)}$ = 4.63, $p < 1 \times 10^{-4}$**		
Waist circumference	$F_{(1,110)}$ = 5466, $p < 1 \times 10^{-4}$	$F_{(1,77)}$ = 1.00, p = 0.32	$F_{(1,110)}$ = 24.18, $p < 1 \times 10^{-4}$	$F_{(2,77)}$ = 0.66, p = 0.52	**$F_{(3,77)}$ = 24.27, $p < 1 \times 10^{-4}$**			$F_{(1,77)}$ = 0.24, p = 0.62	$F_{(1,77)}$ = 0.19, p = 0.67
					$t_{(77)}$ = 0.91, p = 0.36	**$t_{(77)}$ = 4.00, $p = 1 \times 10^{-4}$**	**$t_{(77)}$ = 5.50, $p < 1 \times 10^{-4}$**		
Inflammatory biomarkers									
Interleukin (IL)-2	$F_{(1,109)}$ = 9.84, $p = 2 \times 10^{-3}$	$F_{(1,73)}$ = 0.01, p = 0.92	$F_{(1,109)}$ = 0.95, p = 0.33	$F_{(2,73)}$ = 0.70, p = 0.50	**$F_{(3,73)}$ = 15.85, $p < 1 \times 10^{-4}$**			$F_{(1,73)}$ = 0.28, p = 0.60	$F_{(1,73)}$ = 0.39, p = 0.53
					**$t_{(73)}$ = 4.92, $p < 1 \times 10^{-4}$**	$t_{(73)}$ = 1.61, p = 0.11	**$t_{(73)}$ = 4.31, $p = 1 \times 10^{-4}$**		
IL-4	$F_{(1,109)}$ = 0.0007, p = 0.98	$F_{(1,72)}$ = 0.12, p = 0.73	$F_{(1,109)}$ = 3.68, p = 0.06	$F_{(2,72)}$ = 0.53, p = 0.59	**$F_{(3,72)}$ = 19.80, $p < 1 \times 10^{-4}$**			$F_{(1,72)}$ = 0.004, p = 0.95	$F_{(1,72)}$ = 0.34, p = 0.56
					**$t_{(72)}$ = 5.95, $p < 1 \times 10^{-4}$**	**$t_{(72)}$ = 3.43, $p = 1 \times 10^{-3}$**	**$t_{(72)}$ = 6.52, $p < 1 \times 10^{-4}$**		
IL-6	$F_{(1,109)}$ = 2.51, p = 0.12	$F_{(1,72)}$ = 0.33, p = 0.56	$F_{(1,109)}$ = 0.19, p = 0.67	$F_{(2,72)}$ = 3.83, p = 0.03	$F_{(3,72)}$ = 7.53, $p = 2 \times 10^{-4}$			$F_{(1,72)}$ = 0.15, p = 0.70	$F_{(1,72)}$ = 0.0001, p = 0.99
					**$t_{(72)}$ = 4.22, $p = 1 \times 10^{-4}$**	$t_{(72)}$ = 2.19, p = 0.03	**$t_{(72)}$ = 3.80, $p = 3 \times 10^{-4}$**		
IL-8	$F_{(1,109)}$ = 60.06, $p < 1 \times 10^{-4}$	$F_{(1,72)}$ = 3.83, p = 0.05	$F_{(1,108)}$ = 0.93, p = 0.34	$F_{(2,72)}$ = 0.68, p = 0.51	$F_{(3,72)}$ = 2.70, p = 0.05			$F_{(1,72)}$ = 13.00, $p = 6 \times 10^{-4}$	$F_{(1,72)}$ = 2.88, p = 0.09
					$t_{(72)}$ = 0.65, p = 0.52	$t_{(72)}$ = -0.51, p = 0.62	$t_{(72)}$ = 1.59, p = 0.11		

Continued.

	Intercept	Age	Gender	Smoking status	Disease and Treatment Effect: FEP patients before treatment	FEP patients after 0.6-year treatment	FEP patients after 5.1-year treatment	Time-Diff1	Time-Diff2
	**Effects of Independent Variables on the Dependent Variable (*F*-Value, *p*-Value)**								
					***t*-Value, *p*-Value**				
Inflammatory biomarkers									
IL-10	$F_{(1,109)} = 0.57$, $p = 0.45$	$F_{(1,73)} = 0.72$, $p = 0.40$	$F_{(1,109)} = 0.44$, $p = 0.51$	$F_{(2,73)} = 2.27$, $p = 0.11$	**$F_{(3,73)} = 14.42$, $p < 1 \times 10^{-4}$**			$F_{(1,73)} = 0.64$, $p = 0.43$	$F_{(1,73)} = 0.002$, $p = 0.96$
					$t_{(73)} = 0.80$, $p = 0.43$	$t_{(73)} = 0.72$, $p = 0.48$	**$t_{(73)} = 5.82$, $p < 1 \times 10^{-4}$**		
IL-1A	$F_{(1,109)} = 41.84$, $p < 1 \times 10^{-4}$	$F_{(1,73)} = 0.01$, $p = 0.92$	$F_{(1,109)} = 0.06$, $p = 0.80$	$F_{(2,73)} = 1.31$, $p = 0.28$	**$F_{(3,73)} = 9.21$, $p < 1 \times 10^{-4}$**			$F_{(1,73)} = 0.008$, $p = 0.93$	$F_{(1,73)} = 0.33$, $p = 0.57$
					**$t_{(73)} = 3.44$, $p = 1 \times 10^{-3}$**	$t_{(73)} = 2.00$, $p = 0.05$	**$t_{(73)} = 4.32$, $p < 1 \times 10^{-4}$**		
IL-1B	$F_{(1,108)} = 8.21$, $p = 5 \times 10^{-3}$	$F_{(1,72)} = 6.69$, $p = 0.01$	$F_{(1,108)} = 0.28$, $p = 0.59$	$F_{(2,72)} = 0.94$, $p = 0.40$	$F_{(3,72)} = 2.04$, $p = 0.12$			$F_{(1,72)} = 0.03$, $p = 0.86$	$F_{(1,72)} = 1.14$, $p = 0.29$
					$t_{(72)} = -0.72$, $p = 0.47$	$t_{(72)} = -1.34$, $p = 0.19$	$t_{(72)} = 0.89$, $p = 0.37$		
Interferon gamma (IFN-γ)	$F_{(1,108)} = 53.20$, $p < 1 \times 10^{-4}$	$F_{(1,70)} = 0.19$, $p = 0.66$	$F_{(1,108)} = 0.49$, $p = 0.48$	$F_{(2,70)} = 2.26$, $p = 0.11$	**$F_{(3,70)} = 14.34$, $p < 1 \times 10^{-4}$**			$F_{(1,70)} = 0.47$, $p = 0.49$	$F_{(1,70)} = 3.87$, $p = 0.05$
					$t_{(70)} = 2.64$, $p = 0.01$	$t_{(70)} = -1.52$, $p = 0.13$	**$t_{(70)} = 3.74$, $p = 4 \times 10^{-4}$**		
Tumor necrosis factor-alpha (TNF-α)	$F_{(1,109)} = 24.72$, $p < 1 \times 10^{-4}$	$F_{(1,72)} = 1.65$, $p = 0.20$	$F_{(1,109)} = 3.19$, $p = 0.08$	$F_{(2,72)} = 1.77$, $p = 0.18$	$F_{(3,72)} = 3.73$, $p = 0.01$			$F_{(1,72)} = 1.45$, $p = 0.23$	$F_{(1,72)} = 4.62$, $p = 0.03$
					$t_{(72)} = -0.62$, $p = 0.54$	$t_{(72)} = -0.88$, $p = 0.38$	$t_{(72)} = 1.41$, $p = 0.16$		
Monocyte chemoattractant protein-1 (MCP-1)	$F_{(1,109)} = 318.9$, $p < 1 \times 10^{-4}$	$F_{(1,73)} = 5.95$, $p = 0.02$	$F_{(1,109)} = 1.44$, $p = 0.23$	$F_{(2,73)} = 1.48$, $p = 0.23$	$F_{(3,73)} = 4.03$, $p = 0.01$			$F_{(1,73)} = 0.0001$, $p = 0.99$	$F_{(1,73)} = 4.54$, $p = 0.04$
					$t_{(73)} = -0.02$, $p = 0.98$	$t_{(73)} = 0.42$, $p = 0.67$	$t_{(73)} = 2.48$, $p = 0.02$		
Ferritin	$F_{(1,109)} = 86.73$, $p < 1 \times 10^{-4}$	$F_{(1,74)} = 4.75$, $p = 0.03$	$F_{(1,109)} = 76.03$, $p < 1 \times 10^{-4}$	$F_{(2,74)} = 0.20$, $p = 0.82$	$F_{(3,74)} = 6.07$, $p = 9 \times 10^{-4}$			$F_{(1,74)} = 0.92$, $p = 0.34$	$F_{(1,74)} = 0.39$, $p = 0.54$
					**$t_{(74)} = 3.52$, $p = 7 \times 10^{-4}$**	$t_{(74)} = 1.29$, $p = 0.20$	$t_{(74)} = 1.99$, $p = 0.05$		

Continued.

	Intercept	Age	Gender	Smoking status	Disease and Treatment Effect: FEP patients before treatment	Disease and Treatment Effect: FEP patients after 0.6-year treatment	Disease and Treatment Effect: FEP patients after 5.1-year treatment	Time-Diff1	Time-Diff2
	Effects of Independent Variables on the Dependent Variable (F-Value, p-Value)								
					t-Value, p-Value				
Metabolic biomarkers									
Resistin	$F_{(1,109)}$ = 32.53, p < 1 x 10^{-4}	$F_{(1,74)}$ = 0.17, p = 0.68	$F_{(1,109)}$ = 0.04, p = 0.84	$F_{(2,74)}$ = 0.25, p = 0.78	$F_{(3,74)}$ = 8.96, p = 1 x 10^{-4}			$F_{(1,74)}$ = 0.10, p = 0.76	$F_{(1,74)}$ = 0.54, p = 0.46
					$t_{(74)}$ = 2.68, p = 9 x 10^{-3}	$t_{(74)}$ = 0.77, p = 0.44	$t_{(74)}$ = -2.40, p = 0.02		
Insulin	$F_{(1,108)}$ = 60.79, p < 1 x 10^{-4}	$F_{(1,74)}$ = 1.03, p = 0.31	$F_{(1,108)}$ = 0.18, p = 0.67	$F_{(2,74)}$ = 0.98, p = 0.38	$F_{(3,74)}$ = 4.12, p = 9 x 10^{-3}			$F_{(1,74)}$ = 0.52, p = 0.47	$F_{(1,74)}$ = 1.29, p = 0.26
					$t_{(74)}$ = -1.35, p = 0.18	$t_{(74)}$ = 1.17, p = 0.25	$t_{(74)}$ = -2.15, p = 0.03		
Leptin	$F_{(1,108)}$ = 0.47, p = 0.49	$F_{(1,71)}$ = 0.94, p = 0.33	$F_{(1,108)}$ = 34.62, p < 1 x 10^{-4}	$F_{(2,71)}$ = 0.67, p = 0.51	**$F_{(3,71)}$ = 9.11, p < 1 x 10^{-4}**			$F_{(1,71)}$ = 0.27, p = 0.60	$F_{(1,71)}$ = 0.93, p = 0.34
					$t_{(71)}$ = -0.33, p = 0.74	$t_{(71)}$ = 2.29, p = 0.02	**$t_{(74)}$ = 3.43, p = 1 x 10^{-3}**		
Plasminogen activator inhibitor-1 (PAI-1)	$F_{(1,109)}$ = 68.21, p < 1 x 10^{-4}	$F_{(1,73)}$ = 2.18, p = 0.14	$F_{(1,109)}$ = 0.14, p = 0.71	$F_{(2,73)}$ = 6.10, p = 4 x 10^{-3}	$F_{(3,74)}$ = 8.62, p = 1 x 10^{-4}			$F_{(1,73)}$ = 0.79, p = 0.38	$F_{(1,73)}$ = 1.05, p = 0.31
					$t_{(73)}$ = 2.03, p = 0.05	$t_{(73)}$ = 0.39, p = 0.70	$t_{(73)}$ = -2.86, p = 6 x 10^{-3}		
Acylcarnitines									
C0	$F_{(1,109)}$ = 1008, p < 1 x 10^{-4}	$F_{(1,74)}$ = 0.71, p = 0.40	$F_{(1,109)}$ = 17.05, p = 1 x 10^{-4}	$F_{(2,74)}$ = 0.02, p = 0.98	$F_{(3,74)}$ = 0.66, p = 0.58			$F_{(1,74)}$ = 0.03, p = 0.87	$F_{(1,74)}$ = 0.09, p = 0.76
					$t_{(74)}$ = 0.96, p = 0.34	$t_{(74)}$ = 0.42, p = 0.67	$t_{(74)}$ = 1.26, p = 0.21		
C16	$F_{(1,109)}$ = 381.8, p < 1 x 10^{-4}	$F_{(1,73)}$ = 2.09, p = 0.15	$F_{(1,109)}$ = 3.71, p = 0.06	$F_{(2,73)}$ = 2.75, p = 0.07	**$F_{(3,73)}$ = 10.42, p < 1 x 10^{-4}**			$F_{(1,73)}$ = 1.55, p = 0.22	$F_{(1,73)}$ = 1.32, p = 0.25
					**$t_{(73)}$ = 5.25, p < 1 x 10^{-4}**	$t_{(73)}$ = 1.36, p = 0.18	**$t_{(73)}$ = 3.02, p = 3 x 10^{-3}**		
C16-OH	$F_{(1,109)}$ = 0.04, p = 0.85	$F_{(1,74)}$ = 0.05, p = 0.82	$F_{(1,109)}$ = 0.02, p = 0.89	$F_{(2,74)}$ = 0.52, p = 0.59	$F_{(3,74)}$ = 4.00, p = 0.01			$F_{(1,74)}$ = 0.04, p = 0.84	$F_{(1,74)}$ = 0.40, p = 0.53
					$t_{(74)}$ = 0.66, p = 0.51	$t_{(74)}$ = 0.92, p = 0.36	**$t_{(74)}$ = 3.26, p = 2 x 10^{-3}**		
C16:1	$F_{(1,91)}$ = 557.2, p < 1 x 10^{-4}	$F_{(1,71)}$ = 0.92, p = 0.34	$F_{(1,91)}$ = 0.67, p = 0.41	$F_{(2,71)}$ = 1.14, p = 0.33	$F_{(3,71)}$ = 8.44, p = 1 x 10^{-4}			$F_{(1,71)}$ = 2.30, p = 0.13	$F_{(1,71)}$ = 1.23, p = 0.27
					**$t_{(71)}$ = 3.96, p = 2 x 10^{-4}**	$t_{(71)}$ = 0.50, p = 0.62	**$t_{(71)}$ = 3.40, p = 1 x 10^{-3}**		

Continued.

	Intercept	Age	Gender	Smoking status	Disease and Treatment Effect			Time-Diff1	Time-Diff2
					FEP patients before treatment	FEP patients after 0.6-year treatment	FEP patients after 5.1-year treatment		
	Effects of Independent Variables on the Dependent Variable (F-Value, p-Value)								
	t-Value, p-Value								
Acylcarnitines									
C16:1-OH	$F_{(1,109)} = 0.02$, $p = 0.88$	$F_{(1,74)} = 0.36$, $p = 0.58$	$F_{(1,109)} = 0.08$, $p = 0.77$	$F_{(2,74)} = 0.20$, $p = 0.82$	$F_{(3,74)} = 4.67$, $p = 5 \times 10^{-3}$			$F_{(1,74)} = 0.02$, $p = 0.89$	$F_{(1,74)} = 0.32$, $p = 0.57$
					$t_{(74)} = 1.13$, $p = 0.26$	$t_{(74)} = 1.07$, $p = 0.29$	**$t_{(74)} = 3.56$, $p = 6 \times 10^{-4}$**		
C16:2	$F_{(1,84)} = 535.1$, $p < 1 \times 10^{-4}$	$F_{(1,71)} = 0.20$, $p = 0.66$	$F_{(1,84)} = 0.13$, $p = 0.71$	$F_{(2,71)} = 0.47$, $p = 0.63$	**$F_{(3,71)} = 9.31$, $p < 1 \times 10^{-4}$**			$F_{(1,71)} = 1.78$, $p = 0.19$	$F_{(1,71)} = 0.69$, $p = 0.41$
					$t_{(71)} = 1.20$, $p = 0.24$	$t_{(71)} = -0.30$, $p = 0.76$	**$t_{(71)} = 4.26$, $p = 1 \times 10^{-4}$**		
C16:2-OH	$F_{(1,109)} = 4.90$, $p = 0.03$	$F_{(1,74)} = 1.76$, $p = 0.19$	$F_{(1,109)} = 0.46$, $p = 0.50$	$F_{(2,74)} = 0.70$, $p = 0.50$	$F_{(3,74)} = 6.77$, $p = 4 \times 10^{-4}$			$F_{(1,74)} = 0.03$, $p = 0.87$	$F_{(1,74)} = 0.40$, $p = 0.53$
					**$t_{(74)} = 3.70$, $p = 4 \times 10^{-4}$**	**$t_{(74)} = 4.35$, $p < 1 \times 10^{-4}$**	**$t_{(74)} = 2.97$, $p = 4 \times 10^{-3}$**		
C18	$F_{(1,103)} = 603.2$, $p < 1 \times 10^{-4}$	$F_{(1,74)} = 0.78$, $p = 0.38$	$F_{(1,103)} = 0.45$, $p = 0.50$	$F_{(2,74)} = 0.05$, $p = 0.95$	$F_{(3,74)} = 5.12$, $p = 3 \times 10^{-3}$			$F_{(1,74)} = 0.57$, $p = 0.45$	$F_{(1,74)} = 2.50$, $p = 0.12$
					$t_{(74)} = 2.63$, $p = 0.01$	$t_{(74)} = 0.63$, $p = 0.53$	**$t_{(74)} = 3.04$, $p = 3 \times 10^{-3}$**		
C18:1	$F_{(1,89)} = 245.7$, $p < 1 \times 10^{-4}$	$F_{(1,72)} = 0.66$, $p = 0.42$	$F_{(1,89)} = 0.05$, $p = 0.82$	$F_{(2,72)} = 1.20$, $p = 0.31$	**$F_{(3,72)} = 16.02$, $p < 1 \times 10^{-4}$**			$F_{(1,72)} = 2.53$, $p = 0.12$	$F_{(1,72)} = 0.0005$, $p = 0.98$
					**$t_{(72)} = 4.72$, $p < 1 \times 10^{-4}$**	$t_{(72)} = -0.02$, $p = 0.98$	**$t_{(72)} = 4.01$, $p = 1 \times 10^{-4}$**		
C18:1-OH	$F_{(1,109)} = 8.28$, $p = 5 \times 10^{-3}$	$F_{(1,74)} = 0.11$, $p = 0.74$	$F_{(1,109)} = 1.55$, $p = 0.22$	$F_{(2,74)} = 2.49$, $p = 0.09$	$F_{(3,74)} = 5.49$, $p = 2 \times 10^{-3}$			$F_{(1,74)} = 1.55$, $p = 0.22$	$F_{(1,74)} = 0.12$, $p = 0.73$
					**$t_{(74)} = 3.27$, $p = 2 \times 10^{-3}$**	$t_{(74)} = 2.90$, $p = 5 \times 10^{-3}$	**$t_{(74)} = 3.48$, $p = 9 \times 10^{-4}$**		
C18:2	$F_{(1,99)} = 517.0$ $p < 1 \times 10^{-4}$	$F_{(1,72)} = 1.36$, $p = 0.25$	$F_{(1,99)} = 2.09$, $p = 0.15$	$F_{(2,72)} = 0.10$, $p = 0.91$	**$F_{(3,72)} = 13.85$, $p < 1 \times 10^{-4}$**			$F_{(1,72)} = 1.62$, $p = 0.21$	$F_{(1,72)} = 0.41$, $p = 0.52$
					**$t_{(72)} = 3.87$, $p = 2 \times 10^{-4}$**	$t_{(72)} = 0.16$, $p = 0.87$	**$t_{(72)} = 4.33$, $p < 1 \times 10^{-4}$**		

Continued.

	Intercept	Age	Gender	Smoking status	Disease and Treatment Effect			Time-Diff1	Time-Diff2
					FEP patients before treatment	FEP patients after 0.6-year treatment	FEP patients after 5.1-year treatment		
	Effects of Independent Variables on the Dependent Variable (F-Value, p-Value)								
	t-Value, p-Value								
Acylcarnitines									
C2	$F_{(1,109)} = 57.05$, $p < 1 \times 10^{-4}$	$F_{(1,73)} = 2.77$, $p = 0.10$	$F_{(1,109)} = 3.38$, $p = 0.07$	$F_{(2,73)} = 4.70$, $p = 0.01$	**$F_{(3,73)} = 13.25$, $p < 1 \times 10^{-4}$**			$F_{(1,73)} = 0.004$, $p = 0.95$	$F_{(1,73)} = 0.41$, $p = 0.52$
					$t_{(73)} = -2.02$, $p = 0.05$	$t_{(73)} = -1.36$, $p = 0.18$	**$t_{(73)} = 4.24$, $p = 1 \times 10^{-4}$**		
C3	$F_{(1,109)} = 17.30$, $p = 1 \times 10^{-4}$	$F_{(1,73)} = 1.86$, $p = 0.18$	$F_{(1,109)} = 10.15$, $p = 2 \times 10^{-3}$	$F_{(2,73)} = 0.60$, $p = 0.55$	**$F_{(3,73)} = 43.78$, $p < 1 \times 10^{-4}$**			$F_{(1,73)} = 0.39$, $p = 0.53$	$F_{(1,73)} = 4.57$, $p = 0.04$
					**$t_{(73)} = -5.79$, $p < 1 \times 10^{-4}$**	$t_{(73)} = -1.80$, $p = 0.08$	**$t_{(73)} = 5.27$, $p < 1 \times 10^{-4}$**		
C3-DC(C4-OH)	$F_{(1,109)} = 0.60$, $p = 0.44$	$F_{(1,74)} = 2.98$, $p = 0.09$	$F_{(1,109)} = 1.49$, $p = 0.22$	$F_{(2,74)} = 1.04$, $p = 0.36$	$F_{(3,74)} = 6.17$, $p = 8 \times 10^{-4}$			$F_{(1,74)} = 0.22$, $p = 0.64$	$F_{(1,74)} = 0.005$, $p = 0.94$
					**$t_{(74)} = 4.28$, $p = 1 \times 10^{-4}$**	$t_{(74)} = 2.16$, $p = 0.03$	$t_{(74)} = 1.60$, $p = 0.11$		
C5-OH(C3-DC-M)	$F_{(1,109)} = 13.18$, $p = 4 \times 10^{-4}$	$F_{(1,74)} = 0.04$, $p = 0.83$	$F_{(1,109)} = 1.21$, $p = 0.27$	$F_{(2,74)} = 2.19$, $p = 0.12$	**$F_{(3,74)} = 11.15$, $p < 1 \times 10^{-4}$**			$F_{(1,74)} = 0.04$, $p = 0.84$	$F_{(1,74)} = 0.0002$, $p = 0.99$
					**$t_{(74)} = 4.94$, $p < 1 \times 10^{-4}$**	**$t_{(74)} = 5.06$, $p < 1 \times 10^{-4}$**	**$t_{(74)} = 4.69$, $p < 1 \times 10^{-4}$**		
C3-OH	$F_{(1,109)} = 1.17$, $p = 0.28$	$F_{(1,74)} = 0.09$, $p = 0.77$	$F_{(1,109)} = 0.21$, $p = 0.65$	$F_{(2,74)} = 0.14$, $p = 0.87$	$F_{(3,74)} = 4.77$, $p = 4 \times 10^{-3}$			$F_{(1,74)} = 0.10$, $p = 0.75$	$F_{(1,74)} = 0.06$, $p = 0.81$
					$t_{(74)} = 1.49$, $p = 0.14$	$t_{(74)} = 1.93$, $p = 0.06$	**$t_{(74)} = 3.70$, $p = 4 \times 10^{-4}$**		
C3:1	$F_{(1,109)} = 0.45$, $p = 0.50$	$F_{(1,74)} = 3.28$, $p = 0.07$	$F_{(1,109)} = 0.16$, $p = 0.69$	$F_{(2,74)} = 0.01$, $p = 0.99$	$F_{(3,74)} = 5.44$, $p = 2 \times 10^{-3}$			$F_{(1,74)} = 0.48$, $p = 0.49$	$F_{(1,74)} = 0.007$, $p = 0.93$
					$t_{(74)} = 2.33$, $p = 0.02$	**$t_{(74)} = 3.38$, $p = 1 \times 10^{-3}$**	**$t_{(74)} = 3.49$, $p = 1 \times 10^{-3}$**		
C4	$F_{(1,109)} = 99.47$, $p < 1 \times 10^{-4}$	$F_{(1,73)} = 0.12$, $p = 0.72$	$F_{(1,109)} = 0.58$, $p = 0.45$	$F_{(2,73)} = 0.12$, $p = 0.88$	**$F_{(3,73)} = 35.34$, $p < 1 \times 10^{-4}$**			$F_{(1,73)} = 0.44$, $p = 0.51$	$F_{(1,73)} = 1.25$, $p = 0.27$
					**$t_{(73)} = -5.64$, $p < 1 \times 10^{-4}$**	**$t_{(73)} = -4.43$, $p < 1 \times 10^{-4}$**	**$t_{(73)} = 4.72$, $p < 1 \times 10^{-4}$**		
C4:1	$F_{(1,109)} = 2.17$, $p = 0.14$	$F_{(1,74)} = 2.04$, $p = 0.16$	$F_{(1,109)} = 0.24$, $p = 0.62$	$F_{(2,74)} = 0.01$, $p = 0.99$	**$F_{(3,74)} = 10.53$, $p < 1 \times 10^{-4}$**			$F_{(1,74)} = 0.50$, $p = 0.48$	$F_{(1,74)} = 0.18$, $p = 0.67$
					**$t_{(74)} = 3.40$, $p = 1 \times 10^{-3}$**	**$t_{(74)} = 4.13$, $p = 1 \times 10^{-4}$**	**$t_{(74)} = 5.23$, $p < 1 \times 10^{-4}$**		

Continued.

	Intercept	Age	Gender	Smoking status	Disease and Treatment Effect: FEP patients before treatment	FEP patients after 0.6-year treatment	FEP patients after 5.1-year treatment	Time-Diff1	Time-Diff2
	Effects of Independent Variables on the Dependent Variable (F-Value, p-Value)								
					t-Value, p-Value				
Acylcarnitines									
C5	$F_{(1,109)} = 91.05$, $p < 1 \times 10^{-4}$	$F_{(1,74)} = 1.76$, $p = 0.19$	$F_{(1,109)} = 12.03$, $p = 8 \times 10^{-4}$	$F_{(2,74)} = 0.86$, $p = 0.43$	$F_{(3,74)} = 8.24$, $p = 1 \times 10^{-4}$			$F_{(1,74)} = 0.46$, $p = 0.50$	$F_{(1,74)} = 0.02$, $p = 0.89$
					$t_{(74)} = -2.86$, $p = 6 \times 10^{-3}$	$t_{(74)} = 0.29$, $p = 0.77$	$t_{(74)} = 1.39$, $p = 0.17$		
C5-M-DC	$F_{(1,109)} = 4.36$, $p = 0.04$	$F_{(1,74)} = 0.99$, $p = 0.32$	$F_{(1,109)} = 0.02$, $p = 0.90$	$F_{(2,74)} = 1.18$, $p = 0.31$	$F_{(3,74)} = 3.69$, $p = 0.02$			$F_{(1,74)} = 1.46$, $p = 0.23$	$F_{(1,74)} = 0.16$, $p = 0.69$
					**$t_{(74)} = 3.23$, $p = 2 \times 10^{-3}$**	**$t_{(74)} = 3.04$, $p = 3 \times 10^{-3}$**	$t_{(74)} = 1.54$, $p = 0.13$		
C5:1	$F_{(1,109)} = 0.75$, $p = 0.39$	$F_{(1,74)} = 2.49$, $p = 0.12$	$F_{(1,109)} = 0.18$, $p = 0.67$	$F_{(2,74)} = 0.001$, $p = 0.99$	$F_{(3,74)} = 8.11$, $p = 1 \times 10^{-4}$			$F_{(1,74)} = 0.11$, $p = 0.74$	$F_{(1,74)} = 0.18$, $p = 0.67$
					**$t_{(74)} = 4.06$, $p = 1 \times 10^{-4}$**	**$t_{(74)} = 3.70$, $p = 4 \times 10^{-4}$**	**$t_{(74)} = 4.46$, $p < 1 \times 10^{-4}$**		
C5:1-DC	$F_{(1,109)} = 7.65$, $p = 7 \times 10^{-3}$	$F_{(1,74)} = 0.05$, $p = 0.82$	$F_{(1,109)} = 0.25$, $p = 0.62$	$F_{(2,74)} = 2.63$, $p = 0.08$	$F_{(3,74)} = 5.19$, $p = 3 \times 10^{-3}$			$F_{(1,74)} = 0.04$, $p = 0.83$	$F_{(1,74)} = 0.36$, $p = 0.55$
					**$t_{(74)} = 3.53$, $p = 7 \times 10^{-4}$**	**$t_{(74)} = 3.52$, $p = 8 \times 10^{-4}$**	**$t_{(74)} = 3.14$, $p = 2 \times 10^{-3}$**		
Hexoses									
Hexoses	$F_{(1,109)} = 10690$, $p < 1 \times 10^{-4}$	$F_{(1,73)} = 0.43$, $p = 0.51$	$F_{(1,109)} = 5.81$, $p = 0.02$	$F_{(2,73)} = 0.35$, $p = 0.71$	$F_{(3,73)} = 4.44$, $p = 6 \times 10^{-3}$			$F_{(1,73)} = 5.33$, $p = 0.02$	$F_{(1,73)} = 2.58$, $p = 0.11$
					$t_{(73)} = 0.60$, $p = 0.55$	$t_{(73)} = 0.48$, $p = 0.63$	**$t_{(73)} = 3.40$, $p = 1 \times 10^{-3}$**		
Ratios of acylcarnitines									
(C16+C18)/C0	$F_{(1,103)} = 1737$, $p < 1 \times 10^{-4}$	$F_{(1,73)} = 0.06$, $p = 0.81$	$F_{(1,103)} = 3.93$, $p = 0.05$	$F_{(2,73)} = 1.88$, $p = 0.16$	$F_{(3,73)} = 4.64$, $p = 5 \times 10^{-3}$			$F_{(1,73)} = 1.53$, $p = 0.22$	$F_{(1,73)} = 1.17$, $p = 0.28$
					$t_{(73)} = 3.43$, **$p = 1 \times 10^{-3}$**	$t_{(73)} = 0.73$, $p = 0.47$	$t_{(73)} = 1.85$, $p = 0.07$		
(C16+C18:1)/C2	$F_{(1,89)} = 224.7$, $p < 1 \times 10^{-4}$	$F_{(1,71)} = 5.96$, $p = 0.02$	$F_{(1,89)} = 5.22$, $p = 0.02$	$F_{(2,71)} = 3.33$, $p = 0.04$	**$F_{(3,71)} = 20.81$, $p < 1 \times 10^{-4}$**			$F_{(1,71)} = 0.71$, $p = 0.40$	$F_{(1,71)} = 0.29$, $p = 0.59$
					**$t_{(71)} = 4.18$, $p = 1 \times 10^{-4}$**	$t_{(71)} = 0.72$, $p = 0.47$	$t_{(71)} = -2.56$, $p = 0.01$		
(C2+C3+C4+C5)/C0	$F_{(1,108)} = 50.42$, $p < 1 \times 10^{-4}$	$F_{(1,68)} = 5.13$, $p = 0.03$	$F_{(1,108)} = 6.64$, $p = 0.01$	$F_{(2,68)} = 2.34$, $p = 0.10$	**$F_{(3,68)} = 10.36$, $p < 1 \times 10^{-4}$**			$F_{(1,68)} = 0.14$, $p = 0.71$	$F_{(1,68)} = 0.72$, $p = 0.40$
					$t_{(68)} = -2.81$, $p = 6 \times 10^{-3}$	$t_{(68)} = -1.95$, $p = 0.05$	**$t_{(68)} = 3.00$, $p = 4 \times 10^{-3}$**		
